# Management of anophthalmia, microphthalmia and coloboma in the newborn, shared care between neonatologist and ophthalmologist: a literature review

**DOI:** 10.1186/s13052-025-01882-3

**Published:** 2025-03-05

**Authors:** Monica Russo, Serena Palmeri, Alice Zucconi, Aldo Vagge, Cesare Arioni

**Affiliations:** 1 Operative Unit of Neonatology, IRCCS Ospedale Policlinico San Martino, Genoa, 16132 Italy; 2 Department of Neurosciences, Rehabilitation, Ophthalmology, Genetics and Maternal and Child Sciences (DINOGMI), University of Genoa, Genoa, Italy; 3 IRCCS Ospedale Policlinico San Martino, Genoa, Italy; 4 Clinica Oculistica, DINOGMI, Università di Genova, Genoa, Italy; 5 Fondazione Chiossone, Genoa, Italy

**Keywords:** Anophthalmia, Microphthalmia, Coloboma, Newborn, Congenital eye malformation, Care report

## Abstract

Congenital ocular anomalies significantly contribute to global disability, with 15–20% of infant blindness attributed to these anomalies. This study examined anophthalmia, microphthalmia, and coloboma (AMC) through collaborative neonatology and ophthalmology care.

The global prevalence of AMC varies: anophthalmia at 0.6–4.2 per 100,000 births and microphthalmia at 2–17 per 100,000 births, with a combined prevalence of up to 30 per 100,000. The prevalence of coloboma, alone or associate with other eye defects is 2–19 per 100,000 live births. Anophthalmia and microphthalmia may present as isolated or genetic syndromes, necessitating comprehensive evaluation. AMC etiology encompasses genetic and environmental factors. Chromosomal aberrations and mutations in genes such as *PAX6*, *SOX2*, *OTX2*, and *CHD7* are contributors. Syndromic associations, such as CHARGE (heart defect, atresia choanae, retarded growth and development, genital hypoplasia, ear anomalies/deafness) syndrome, underscore the complexity of this syndrome. Early AMC diagnosis is pivotal for timely intervention. This work provides a literature review offering insights for effective management and genetic counseling in a pediatric context.

**Supplementary Information:**

The online version contains supplementary material available at 10.1186/s13052-025-01882-3.

## Introduction

Congenital ocular anomalies are a significant cause of disability worldwide and are estimated to be responsible for approximately 15–20% of blindness and severe visual impairment in infancy [1]. 

Anophthalmia is defined as the complete absence of the eye globe in the orbit.

Microphthalmia refers to an underdeveloped eye of subnormal size, usually defined in terms of corneal diameter or axial length [2, 3]. An eye is termed microphthalmic when the axial diameter is < 16 mm at birth, < 18.5–19 mm in adults (and in children aged > 1 year) [1, 4]. 

The term coloboma describes a segmental ocular defect in any ocular tissue consistent with failure of closure of the embryonal fissure. The embryonal fissure is located inferonasally and extends into a cleft on the lower surface of the optic stalk. Hence, embryonal fissure-related problems can involve, in addition to the choroid and retina, the optic disc posteriorly and the iris and ciliary body anteriorly.

“Typical” coloboma refers to a defect in the inferior/inferonasal part of the fundus that can be clearly attributed to a defect in the closure of the embryonal fissure. There is a rare subgroup of colobomas, referred to as “atypical,” which are located in other parts of the eye (nasal, temporal, or superior), and their pathogenesis has not yet been clarified. Atypical coloboma appears to be sporadic, as no familial cases have been identified yet [4–6]. 

Early detection of these malformations is important for providing early and more effective treatment and rehabilitation strategies and for providing genetic counseling, if appropriate.

We will provide a literature review on major structural eye malformations, such as anophthalmia, microphthalmia, and coloboma (AMC), along with appropriate multidisciplinary management in a pediatric contest.

## Epidemiology

The prevalence of the main congenital anomalies of the eyeball (microphthalmos, anophthalmos, and coloboma) results in wide variability among different geographical areas. The birth prevalence of anophthalmia ranges from 0.6 to 4.2 per 100,000 births, from 2 to 17 per 100,000 births for microphthalmia, while the combined birth incidence has been reported to reach 30 per 100,000 people [1, 2, 7–9]. 

The prevalence of coloboma, alone or associate to other eye defects is 2–19 per 100,000 live births [5]. 

Anophthalmia and microphthalmia are bilateral in most cases, except for isolated microphthalmia, which is usually unilateral [2]. Both anophthalmia and microphthalmia can be isolated or associated with other ocular malformations or organ disease or can be part of a genetic syndrome. The most common associated congenital ocular anomaly is a coloboma in the same or contralateral eye [10]. The risk factors for these malformations suggested by epidemiological studies are maternal age over 40 years, multiple pregnancies, low birth weight and low gestational age [2].

## Relevant embryology

Eye development occurs in human embryos between approximately the third and tenth weeks of gestation. Ocular tissues are mesodermal and ectodermal in origin. The retina, ciliary body, optic nerves, and iris derive from the neuroepithelium. The lens, eyelid, and corneal epithelium develop from the surface ectoderm. The sclera, blood vessels, ocular muscles, vitreous, corneal endothelium, and stroma develop from the extracellular mesenchyme [5]. The homeobox gene *PAX6* is essential for the initiation of the process, which begins with evagination of the optic grooves in the medial anterior neural plate. During the fourth week of pregnancy, upon neural tube closure, the optic grooves are transformed into optic vesicles through the forebrain. The optic vesicles invaginate to form a double-layered optic cup, the inner layer of which develops into the retinal pigment epithelium, while the outer layer forms the retinal pigment epithelium (RPE). The iris and ciliary body develop from the middle part of the optic cup. As the optic cup invaginates, the surface ectoderm forms the lens placode and then develops into the lens vesicle [11, 12]. A ventral invagination along the optic cup and optic stalk termed the embryonal, choroidal, fetal fissure permits the mesenchyme to enter the optic cup and form blood vessels. This fissure closes normally after 5–7 weeks of gestation (at approximately the 17-mm stage) [12]. Several transcription factors, such as *PAX6*, *SIX3*, *LHX2*, and *RAX*, are needed at each stage of eye development. Extrinsic factors, including members of the transforming growth factor (TGFβ), fibroblast growth factor (FGF), sonic hedgehog (SHH), and WNT signaling pathway, are important regulators of ocular embryogenesis [4, 11]. 

## Etiology and genetics

The etiology of AMC is complex, and both genetic and environmental factors are involved. Genetic contributions are significant, and multiple genes have been identified in association with AMC. AMC can be caused by numerical chromosomal defects (duplications, deletions, or translocations) as well as by mutations in selected genes. Chromosomal aberrations are typically associated with characteristic syndromes, such as trisomy 13 (Patau syndrome) and trisomy 18 (Edwards syndrome), or with systemic anomalies in addition to ocular malformation. Rare but equally significant defects should be considered on a case-by-case basis [13, 14]. 

Monogenic causes include variants in many single genes, including *PAX6*, *SOX2*, *OTX2* and *CHD7* [2, 15]. The mutations can be de novo or inherited. The main reported chromosomal abnormalities and single gene mutations associated with AMC are listed in Tables 1 and 2, respectively.

**Table 1:** Chromosomal abnormalities associated with anophthalmia/microphthalmia/coloboma [2, 15]

Chromosomal abnormality	AMC phenotype	Syndrome/associated phenotype
3q21-qter duplication	Microphthalmia, coloboma	Pre- and postnatal growth deficiency, microcephaly, CNS anomalies, hypertrichosis, heart defects, chest deformities, genitourinary abnormalities.
4p16.3 deletion	Microphthalmia, iris coloboma	Wolf-Hirschhorn syndrome: growth deficiency, microcephaly, epilepsy, intellectual disability, craniofacial malformations (“Greek warrior helmet” appearance, broad bridge of the nose, micrognathia, cleft lip and/or palate), ear malformations, cochlear deafness.
Mosaic Trisomy 9	Microphthalmia, iris coloboma	Craniofacial malformations, heart defects, feeding and respiratory difficulties, cryptorchidism, hip dysplasia, seizures, and developmental delay
10q duplication	Iris coloboma	Prenatal growth deficiency, microcephaly, developmental delay, camptodactyly, syndactyly, heart defects
Trisomy 13	Anophthalmia, microphthalmia, coloboma	Patau syndrome (holoprosencephaly, microcephaly, retinal dysplasia, cyclopia, cleft lip/palate, heart defects, genital abnormalities)
Trisomy 18	Microphthalmia, iris coloboma	Edwards syndrome (polyhydramnios, single umbilical artery, small placenta, low foetal activity, hypotonia followed by hypertonia, delayed psychomotor development, cranio-facial malformations, heart defects)
Triploidy	Anophthalmia, microphathalmia, coloboma	Large placenta with hydatidiform changes, growth deficiency, syndactyly, CNS anomalies (holoprosencephaly, hydrocephalus myelomeningocele), heart defects, renal malformations

**Table 2:** Gene mutations associated with anophthalmia/microphthalmia/coloboma [2, 16–20, 22–25, 28]

Gene	OMIM	Inheritance	AMC phenotype	Syndrome/associated phenotype
ABCB6	605,452	AD	Microphthalmia, Coloboma of iris, retina and choroid	-
ACTB	102,630	AD	Coloboma of iris and retina	Baraitser-Winter syndrome 1 (craniofacial malformations, CNS anomalies, ocular anomalies, intellectual disability, growth deficiency)
ACTG1	102,560	AD	Coloboma of iris and choroid	Baraitser-Winter syndrome 2 (craniofacial malformations, CNS anomalies, ocular anomalies, growth deficiency)
ALDH1A3	600,463	AR	Microphthalmia, retinal coloboma	-
ALG3	608,750	AR	Coloboma of iris	Congenital disorder of glycosylation, type Id (CNS anomalies, ocular anomalies, facial dysmorphism)
ALX1	601,527	AR	Microphthalmia, coloboma of iris and eye lid	Frontonasal dysplasia
ATOH7	609,875	AR	Microphthalmia	Persistent hyperplastic primary vitreous
BCOR	300,485	XLD	Microphthalmia, Coloboma of iris, choroid and optic nerve	Lenz microphthalmia syndrome (ocular anomalies, dental anomalies, CNA anomalies, heart defects)
BMP4	112,262	AD	Microphthalmia, anophthalmia, coloboma	Orofacial cleft
BMP7	112,267	AD	Microphthalmia, anophthalmia, Coloboma of retina, choroid and optic nerve	Developmental delay, deafness, scoliosis, and cleft palate
CHD7	608,892	AD	Coloboma of iris, retina, choroid and optic nerve, eye lid (rarely)	CHARGE syndrome (coloboma, heart defects, choanal atresia, growth retardation, genital abnormalities, ear abnormalities)
CREBBP	600,140	AD/del	Microphthalmia, coloboma of iris, choroid and retina	Rubinstein-Taybi syndrome (mental retardation, postnatal growth deficiency, microcephaly, broad thumbs and halluces, facial dysmorphism)
CRIM1	606,189	AD	Coloboma of iris, retina, choroid and optic nerve	-
CRYAA	123,580	AD	Coloboma of iris	Cataract
CRYBA4	123,631	AD	Microphthalmia	Cataract
DPYD	612,779	AR	Microphthalmia, coloboma of iris and choroid	Dihydropyrimidine dehydrogenase deficiency
FAT1	600,976	AR	Microphthalmia, coloboma of choroid and retina	Glomerulonephropathy, syndactyly
FBN1	134,797	AR	Coloboma of lens; rarely iris, retina and optic disk coloboma	Marfan syndrome (skeletal anomalies, ocular anomalies, cardiovascular anomalies)
FBN2	612,570	AD	Coloboma of retina and choroid	Congenital contractural arachnodactyly
FGFR1	136,350	Somatic Mosaicism	Microphthalmia, anophthalmia, coloboma of iris and eye lid	Encephalocraniocutaneous lipomatosis (ocular anomalies, skin lesions, central nervous system anomalies)
FGFR2	176,943	AD	Coloboma of iris	Multiple
FOXE3	601,094	AD, AR	Microphthalmia, Coloboma of iris, retina and optic dis	Anterior segment dysgenesis, Cataract, aniridia
FREM1	608,944	AR	Anophthalmia, microphthalmia and coloboma of upper eyelid	Manitoba oculotrichoanal syndrome (MOTA) syndrome (ocular anomalies, aberrant scalp hairline, anal malformations)
FZD5	601,723	AD	Microphthalmia, Coloboma of iris, retina and choroid	-
GDF6	601,147	AD AR	Microphthalmia, Coloboma of iris, retina, choroid and optic nerve	Klippel-Feil Syndrome 1 (facial asimmetry, cleft palate, deafness, skeletal anomalies), Leber congenital amaurosis 17
GJA8	600,897	AD	Microphthalmia	Congenital cataracts
HMGB3	300,193	XL	Microphthalmia, Coloboma of iris, retina and choroid	Microcephaly, short stature, and intellectual disability
HMX1	142,992	AR	Coloboma of iris, retina and choroid	Oculoauricular syndrome (ocular anomalies, ear anomalies)
IGBP1	300,139	XLR	Coloboma of iris and optic nerve	Corpus callosum defect, intellectual disability, micrognathia
IPO13	610,411	AR	Microphthalmia, coloboma of iris, cataract	-
KCTD1	613,420	AD	Coloboma of iris and eye lid	Scalp-ear-nipple syndrome (aplasia cutis congenita of the scalp, breast anomalies, ear anomalies)
KMT2D	602,113	AD	Coloboma of iris, retina, choroid and optic nerve	Kabuki syndrome (intellectual disability, postnatal growth deficiency, skeletal anomalies, facial dysmorphism)
LCP1	153,430	AD	Coloboma of iris and choroid	-
LRP2	600,073	AR	Coloboma of iris	Donnai-Barrow syndrome (facial dysmorfism, ocular anomalies, sensorineural hearing loss, proteinuria)
MAB21L2	604,357	AD, AR	Microphthalmia, Anophthalmia, Coloboma of iris and retina	Rhizomelic skeletal dysplasia
MAF	177,075	AD	Coloboma of iris	Cataract
MITF	156,845	AR	Microphthalmia, coloboma	COMMAD syndrome (coloboma, osteopetrosis, microphthalmia, macrocephaly, albinism, and deafness)
MKS1	609,883	AR	Microphthalmia, coloboma of iris	Meckel-Gruber syndrome (cystic renal disease, CNS malformations, hepatic abnormalities)
GLI2	600,037	AD	Microphthalmia, anophthalmia	Retinal dystrophy, pituitary dysfunction
PAX6	607,108	AD	Microphthalmia, anophthalmia, Coloboma of iris, retina, choroid and optic nerve	Aniridia, Morning glory disc anomaly, Peter’s Anomaly, Anterior segment dysgenesis, Cataract with late onset corneal dystrophy, Foveal hypoplasia, Keratitis, Optic nerve hypoplasia
PIGL	605,947	AR	Coloboma of retina and choroid	CHIME syndrome (colobomas, congenital heart defects, migratory ichthyosiform dermatosis, mental retardation, ear anomalies)
PITX2	601,542	AD	Microphthalmia, coloboma of iris	Axenfeld-Rieger syndrome type 1 (ocular anomalies, maxillary hypoplasia, hypodontia, umbilical defect)
POMT1	607,423	AR	Microphthalmia, coloboma	Muscular dystrophy-dystroglycanopathy, type A, 1 (congenital muscular dystrophy, ocular anomalies, CNS anomalies, intellectual disability)
PTCH1	601,309	AD/Sporadic	Coloboma of iris	Holoprosencephaly, Basal cell nevus syndrome
PTPN11	176,876		Coloboma of iris, retina and optic nerve	Noonan syndrome (growth deficiency, facial dysmorphism, heart defects)
RAB3GAP1	602,536	AR	Microphthalmia, anophthalmia, coloboma of iris, choroid, retina, optic nerve	Warburg Micro syndrome-1 (ocular anomalies, microcephaly, corpus callosum hypoplasia, intellectual disability, spastic diplegia, hypogonadism)
RARB	180,220	AR/AD	Microphthalmia	Diaphragmatic hernia, pulmonary hypoplasia, heart defects
RAX	601,881	AR	Anophthalmia, microphthalmia, coloboma of optic nerve	Variable presence of midline defects, including cleft lip and palate, absence of frontal and/or sphenoidal sinuses, and absent pituitary gland
SALL4	607,343	AD	Microphthalmia, coloboma of iris, choroid and optic nerve	Duane-radial ray syndrome or acrorenoocular syndrome (upper limb anomalies, ocular anomalies, renal anomalies)
SHH	60,072	AD	Microphthalmia, Coloboma of retina, choroid, iris and retina	Holoprosencephaly
SIX3	603,714	AD	Microphthalmia, coloboma of iris, retina, choroid and macula	Holoprosencephaly 2, Schizencephaly
SMCHD1	614,982	AD	Microphtalmia, arhinia	Bosma Arhinia Microphthalmia Syndrome (BAMS)
SMOC1	608,488	AR	Microphthalmia, Coloboma of retina and limb anomalies	Limb anomalies
SMO	601,500	AD	Microphthalmia, coloboma of iris	Curry-Jones syndrome (cutaneous anomalies including patchy skin lesions and syndactyly, hair anoamlies including ectopic patch of facial hair, cerebral malformations, unicoronal craniosynostosis, gastrointestinal anomalies)
SOX2	184,429	AD	Anophthalmia, microphthalmia, coloboma of iris, retina and choroid	Optic nerve hypoplasia, CNS anomalies
SOX3	313,430	XL (germline mosaicism)	Microphthalmia, Coloboma	Panhypopituitarism, intellectual disability
SPINT2	605,124	AR	Coloboma of optic nerve	Congenital secretory sodium diarrhea
STRA6	610,745	AR	Microphthalmia, anophthalmia, coloboma	-
TENM3/ ODZ3	610,083	AR	Microphthalmia, iris coloboma	-
TFAP2A	107,580	AD	Microphthalmia, coloboma of iris, choroid and optic nerve	Branchiooculofacial syndrome (branchial cleft sinus defects, ocular anomalies, facial dysmorphism)
TGDS	616,146	XLR	Coloboma of iris	Catel-Manzke syndrome (Pierre Robin anomaly, bilateral hyperphalangy, clinodactyly)
VAX1	604,294	AR	Microphthalmia	Small optic nerves, orofacial clefting, agenesis of corpus callosum
VSX2	142,993	AR	Microphthalmia, anophthalmia, iris coloboma	Cataracts
WDR11	606,417	AD	Coloboma of iris	Hypogonadotropic hypogonadism with or without anosmia
WASHC5	610,657	AR	Coloboma of iris and retina	Ritscher-Schinzel syndrome or 3 C syndrome (craniofacial abnormalities, congenital heart defects, cerebellar brain malformations)
ZEB2	605,802	AD	Microphthalmia, coloboma of iris and retina	Mowat-Wilson syndrome (ocular anomalies, CNS anomalies, heart defects)

In severe bilateral cases of anophthalmia and microphthalmia, a genetic cause has been identified in approximately 80% of cases, with de novo heterozygous loss-of-function point mutations in *SOX2* being the most common, accounting for 10–20% of cases [2, 16]. 

*SOX2*-associated ocular malformations, including anophthalmia, microphthalmia, sclerocornea, cataracts, persistent hyperplastic primary vitreous and optic disc dysplasia, are variable in type but are most often bilateral and severe. The phenotype of “SOX2 anophthalmia syndrome” includes extraocular features such as learning disabilities, facial dysmorphisms, postnatal growth failure, esophageal atresia with or without trachea-esophageal fistula and urogenital anomalies [17, 18]. 

Although, as mentioned before, the *PAX6* gene is embryologically involved in eye development, its mutations are rare causes of anophthalmia/microphthalmia. Heterozygous loss-of-function (LOF) mutations of this gene, located on chromosome 11p13, are typically associated with aniridia, a congenital panocular malformation characterized by variable gravity [2]. 

Mutations in *OTX2*, *RAX* and *CHX10*, three genes expressed in the retina, are reportedly associated with anophthalmia/microphthalmia, possibly causing failure of retinal differentiation [2]. Heterozygous loss of function mutations in *OTX2* on chromosome 14q22 cause a wide variety of ocular anomalies, ranging from anophthalmia/microphthalmia to retinal defects, eventually associated with CNS malformations [19]. Mutations in *RAX*, located on chromosome 18q21.32, account for approximately 2% of inherited anophthalmia/microphthalmia [20]. Another 2% of isolated microphthalmia is attributed to mutations in the *CHX10* gene on chromosome 14q24.3, with autosomal recessive inheritance [21]. Mutations in MSCHD1 gene have been reported in individuals with eye hypoplasia and nose malformations [22, 23]. 

AMC has also been described in the context of a genetic syndrome. Mutations in the *GLI2* gene were first reported in association with holoprosencephaly and polydactyly; subsequently, anophthalmia and orbital anomalies have also been incorporated into the phenotype [24]. Mutations in the *STRA6* gene cause a variable syndromic phenotype that includes anophthalmia, congenital heart defects and diaphragmatic hernia, pulmonary abnormalities and intellectual disability [25]. 

The most common genetic syndrome associated with coloboma is CHARGE syndrome, an acronym that describes its wide range of clinical features, including coloboma, heart defects, choanal atresia, retardation (of growth and/or development), genitourinary malformation and ear abnormalities. In CHARGE syndrome, almost all patients have intrinsic ophthalmic defects in at least one eye. These include optic nerve/retinochoroidal coloboma, microphthalmia, cataract, and iris coloboma [26]. Currently, the only gene known to be implicated in CHARGE syndrome is *CHD7*, located on 8q12, which regulates the transcription of other tissue-specific targets and possibly disrupts neural crest migration when mutated [27]. 

Another rare syndrome which can be associated with ocular defects is Cat Eye syndrome, named after the characteristic elongated shape of the pupil that may be present in this condition. The three most common features are the symptom triad of preauricular anomalies, anal atresia, and iris coloboma, though there is a very broad phenotypic range. It is typically caused by a partial tetrasomy of chromosome 22, which arises from a supernumerary dicentric marker chromosome featuring satellite structures at both ends and an inverted duplication of chromosome 22 [28]. 

Environmental factors are also implicated in the etiology of AMC. The strongest evidence of gestational-acquired infections is associated with syphilis, rubella, varicella, toxoplasmosis, cytomegalovirus, and other viruses, such as parvovirus B19, influenza virus, and coxsackie A9 [2, 29–31]. The principal environmental causes of congenital coloboma are reported in Table 3 [32]. 

**Table 3:** Environmental causes of congenital coloboma [2, 4, 29–33]

Vitamine A deficiency	Human and animal reports
Toxicity and Exposures Alcohol	Human and animal reports
Methimazole	Human and animal reports
Radiation	Human and animal reports
Mycophenolate Mofetil (MMF)	Human and animal reports
Maternal Thyroid Disease	Human reports
Maternal Diabetes	Human reports
Assisted Reproductive Technologies	Human reports
Hydroxyethylrutoside (HER)	Human reports
Anticonvulsants	Human reports
Lysergic Acid Diethylamide (LSD)	Human reports
Thalidomide	Human reports
Cytomegalovirus Infection (CMV)	Human reports
Toxoplasmosis	Human reports
Zika Virus	Human reports
Vitamine E deficiency	Animal reports
Folate deficiency	Animal reports
Nickel excess	Animal reports
Saccharine	Animal reports
Thiourea	Animal reports
Hyperthermia	Animal reports
Synthetic Cannabinoids	Animal reports

Several noninfectious causes have been proposed, including vitamin A deficiency, maternal diabetes, hypothyroidism, maternal consumption of drugs such as thalidomide, carbamazepine, idantoin, warfarin, exposure to X-rays, and hyperthermia [2, 4]. 

Notably, perinatal alcohol exposure can cause various ocular defects. Visible eye abnormalities in fetal alcohol syndrome include shortened and horizontal palpebral fissures, telecanthus, epicanthus, and blepharoptosis. Strabismus has also been reported. Intrinsic eye structure defects, which indicate early exposure to these teratogens, include microphthalmia, buphthalmos, iris and uveal coloboma, and retinal or vitreous malformations [33]. 

## Diagnosis and management

Usually, congenital ocular malformation is initially suspected by neonatologists based on clinical examination. Then, diagnosis and management require ophthalmological assessment and imaging. A comprehensive family and medical history, physical examination, instrumental and laboratory tests and genetic testing will be needed to establish a specific etiology, and then to provide appropriate counselling to families.

### Pediatric examination

Clinical examination of the newborn by the neonatologist usually first raises the diagnostic suspicion at birth: inspection and palpation of the eye globe through the lids to confirm its presence and obtain an estimate of the ocular size is essential. The majority of cases are identified because of visible eye anomalies, such as a small eyeball, gross nystagmus, strabismus or an obvious iris coloboma [4, 34, 35]. 

The red-reflex test (RRT) is a valuable tool for pediatricians for screening ocular anomalies: any factor that impedes, blocks, or changes this path of the light will result in an abnormal RRT. High and/or asymmetrical refractive defects and ocular misalignment (strabismus) can alter RRT. Anophthalmia and microphthalmia could determine asymmetrical or absent RRT [34, 36]. 

Specifically, gross nystagmus and strabismus may subtend different and wide etiologies including neurological diseases, rather than ocular malformations.

Congenital nystagmus can be linked to several ocular and neurological conditions, or idiopathic. Common causes include Leber congenital amaurosis, albinism, aniridia, achromatopsia, and optic nerve hypoplasia. Other conditions such as optic atrophy, bilateral congenital cataracts, and congenital stationary night blindness may also contribute to its development. A thorough assessment is essential to identify the underlying cause in affected individuals and guide appropriate management strategies.

Congenital strabismus in newborns can be caused by damage to the eye muscles or the nerves that innervate them. Such damage may be associated with conditions like cerebral palsy or, less commonly, acute vascular injury. The clinician should carefully exclude any trauma that may have occurred during the peripartum period [37]. 

A complete physical examination is recommended to identify any associated dysmorphic features or malformations.

### Ophthalmological assessment

Once the suspicion of a congenital ocular anomaly has been established, a specialist ophthalmological assessment is needed to study the transparency of the dioptric apparatus, examine the fundus, and measure the corneal diameters and axial length.

Anophthalmia can be a difficult diagnosis to make by clinical examination. In some cases, with no clinical evidence of a globe or ocular tissue, residual neuroectoderm was demonstrated on histological samples; hence, the use of terms such as “clinical anophthalmia” and “extreme microphthalmia” may refer to a phenotypic spectrum ranging from anophthalmia to microphthalmia [2]. 

Microphthalmia is diagnosed by measuring the corneal diameter, which ranges from 9 to 10.5 mm in neonates and 10.5–12 mm in adults. Nevertheless, corneal diameter cannot always be used as a surrogate marker of eye dimension, as normal-sized eyeballs can also present with microcornea. An ophthalmologist can more precisely measure the axial length of the eye via ultrasonography [2]. 

The diagnosis of a coloboma and its extent require accurate evaluation of both the anterior and posterior segments of the eye. Iris involvement is often observed in association with fundus coloboma. A complete iris typical coloboma appears as an inferonasal defect that merges with the pupil in the shape of a keyhole, while an incomplete iris coloboma can be seen as a notch in the inferior pupillary border or a defect in the pigment epithelium or heterochromia. In contrast to traumatic iris defects, the margins of a coloboma are smooth. A lens coloboma can be seen in a dilated eye as the equator of the lens flattens in an area without zonular fibers. In fundus coloboma, the choroid and retinal pigment epithelium are absent and appear as a white area of bare sclera with occasional spots of pigment deposition at the junction with the normal retina; the border can be smooth or scalloped. Its extent is variable, as it can reach and invade the periphery or be restricted to islands along a line joining disc with an inferior/inferonasal periphery. Bridge coloboma is a term used to describe two islands of colobomas interspersed with a normal retina. The examiner should evaluate for retinal detachment (RD), as patients with colobomas have an increased risk of RD during their lifetime [4]. 

A thorough examination of the family history, focusing on the presence of ocular anomalies, along with ophthalmological assessments of both parents, should be conducted.

### Imaging

Eye ultrasound is recommended for accurate determination of the axial length of the globe and for evaluation of internal ocular structures [2]. 

Computed tomography (CT) and magnetic resonance imaging (MRI) of the orbits can aid clinicians in the diagnosis of anophthalmia, demonstrating the absence of ocular tissue within the orbit, which is usually associated with reduced orbital dimensions; residual optic nerve neural tissue and extraocular muscles are variable. The microphthalmic eye appears on CT and MRI scans as an eye globe of reduced dimensions with normal density/signal intensity of lens and vitreous, usually in a smaller orbit. In addition, orbital CT or MRI scan may show the presence of a cyst posterior to the eyeball which can sometimes be associated with microphthalmos [2, 38]. 

### Further investigations

Further investigations may be needed depending on the clinical picture. Brain MRI allows the study of the intracerebral optic pathways (optic nerves, optic chiasm, optic tracts, optic radiations) and potentially associated cerebral malformations, such as septo-optic dysplasia, a rare congenital disorder characterized by the triad of: (a) optic nerve hypoplasia, (b) midline developmental defects including agenesis of the septum pellucidum, agenesis or dysgenesis of the corpus callosum, or both, and (c) anomalies of the hypothalamic-pituitary axis. This malformation is reported in association with bilateral anophthalmia/microphthalmia [39]. 

In a study conducted by Huynh et al. on 99 patients with apparently isolated uveal coloboma, abnormal findings were detected via echocardiography (53%, 10 of 19 patients who underwent echocardiography; ventral septal defects were the most prevalent), brain MRI (17%, 5 of 29 patients), audiology testing (17%, 13 of 75 patients), spine X-ray (13%, 10 of 77 patients) and kidney US (7%, 5 of 72 patients). Therefore, the authors suggest a protocol for the evaluation of seemingly isolated uveal colobomas, which includes physical examination, baseline audiology assessment, renal US and spine radiography [40]. 

## Prenatal diagnosis

Prenatal diagnosis of AMC has become increasingly important for early intervention and management.

### Imaging in prenatal diagnosis

The prenatal diagnosis of AMC is increasingly important for early intervention and management. Various imaging techniques, such as ultrasound and fetal MRI, are used to detect coloboma-related anomalies during pregnancy. Intrauterine MRI can identify eye malformations like anophthalmia and microphthalmia, as well as nervous tissue abnormalities associated with coloboma. However, isolated prenatal diagnoses of coloboma using MRI have been reported [41, 42]. 

The use of ultrasound for diagnosis of fetal ocular defects was first described in 1991 [43]. Orbital imaging should begin at 12 weeks’ gestation, with detailed eye examinations playing a crucial role in diagnosing microphthalmia or anophthalmia. This helps with genetic counseling, postnatal care planning, and parental preparation, including discussions on pregnancy termination. Two-dimensional ultrasound may show an absence of the eye globe and lens, while three-dimensional reverse-face imaging, as described by Araujo et al. in 2012, can confirm the diagnosis and reveal additional features, such as sunken eyelids and hypoplastic orbits, even when fetal head position interferes with 2D imaging [44]. Three-dimensional reverse-face imaging may reveal valuable additional sonographic features, including sunken eyelids and small or hypoplastic orbit on the affected side and may be considered even superior to 2D when fetal head is deviated. The absence of a lens (aphakia) or the presence of hyaloid arteries should alert clinicians to potential eye abnormalities. These conditions may be associated with complications such as orbital cysts or hemangiomas, which can hinder accurate evaluation [45]. 

Fetal MRI complements ultrasound by providing detailed insights into central nervous system malformations and confirming the absence of eye tissue, optic nerves, and extraocular muscles in cases of anophthalmia [46]. 

### Molecular prenatal diagnosis

Invasive prenatal diagnostics offer the opportunity for genetic diagnosis before birth through the analysis of fetal cells collected via procedures such as amniocentesis (usually after 14 weeks of gestation) or chorionic villus sampling (from 10 to 12 weeks of gestation). Among the applicable cytogenetic tests are karyotyping, chromosomal microarray (including SNP array and CGH array), and targeted next generation sequencing (NGS) panels. In case of suspect of a genetic condition, postnatal cord blood testing is also recommended. Non invasive prenatal investigations include genetic testing using cell-free fetal DNA (cffDNA) present in maternal blood [46, 47]. The prenatal genetic workup should be planned in consultation with a geneticist, considering the fetus’s malformative characteristics and family history.

## Treatment

In managing microphthalmia/anophthalmia, the primary aim is to support optimal visual function development, depending on the disease’s severity and the ocular structures’ integrity and developmental capacity.

The treatment of congenital coloboma includes various medical and surgical interventions. Medical treatment primarily involves addressing associated vision impairments and promoting visual development in affected individuals. This can be achieved through the early prescription of corrective lenses, occlusion therapy to improve binocular vision, and regular monitoring by ophthalmologists specializing in pediatric eye care.

The proper development of orbital cavity and craniofacial conformation is achieved whenever needed through surgically assisted cavity expansion with socket expansion and eye conformers. Given the often genetic etiology of this condition, genetic counseling is essential, allowing for coordinated referrals to relevant specialists for comprehensive care [2, 48–50]. 

Surgical management for the craniofacial development is pivotal for the correct psychological development of young patients, as those affected by forms of hemifacial microsomia are usually affected by higher risk of behavior problems, social difficulties and less acceptance. Therefore, early implementation of eye prothestetics and eye conformers is recommended [51, 52]. 

It is important to note that the appropriateness and timing of surgical interventions depend on individual cases and should be determined by a multidisciplinary team comprising ophthalmologists, geneticists, and pediatric surgeons [1, 9]. 

## Communication with family and support

Since the neonatologist is the first doctor to examine the baby after birth, it is essential to establish effective communication with the family and to convey any suspected and/or confirmed diagnoses under the best possible conditions. The presence of the various professionals involved in managing the clinical case would be desirable, as would avoiding a short duration for the first meeting or an uncomfortable environment [53]. 

## Conclusion

The diagnosis of congenital coloboma, whether isolated or associated with additional clinical issues, is challenging due to the wide range of environmental and genetic causes. Recent advancements in prenatal radiological diagnostics and genetic counseling have enabled early identification of familial cases, promoting timely intervention. However, for all other cases, an early diagnosis through prompt examination of the newborn’s eyes by a neonatologist and a coordinated approach between the neonatologist and ophthalmologist remain essential. Neonatologists can coordinate the multidisciplinary input needed to offer optimal care for newborns [54]. 

## Electronic supplementary material

Below is the link to the electronic supplementary material.

Supplementary Material 1

Supplementary Material 2

Supplementary Material 3

## Data Availability

not applicable.

## Abbreviations

AMC: Anophthalmia, microphthalmia, and coloboma
CT: Computed tomography
FGF: Fibroblast growth factor
MRI: Magnetic resonance imaging
NGS: Next-generation sequencing
OAEs: Otoacoustic emissions
RD: Retinal detachment
RRT: Red-reflex test
RT-PCR: Real-time polymerase chain reaction
SHH: Sonic hedgehog
TGFβ: Transforming growth factor
