# Supplementary material for: Management of anophthalmia, microphthalmia and coloboma in the newborn, shared care between neonatologist and ophthalmologist: a literature review
Source: Ital J Pediatr. 2025 Mar 5;51:65. doi: 10.1186/s13052-025-01882-3 (PMC11881466; doi:10.1186/s13052-025-01882-3)
Supplement: Supplementary file 1 — Supplementary Material 1 [file 13052_2025_1882_MOESM1_ESM.pdf]

Dear Editors and Reviewers,

We thank you again for the opportunity to revise our manuscript ITJP-D-24-00196, titled “Management of anophthalmia, microphthalmia and coloboma in the newborn, shared care between neonatologist and ophthalmologist: a literature review”.

Below, we provide our responses to the suggested revisions (in bold). Attached, you will find the revised manuscript as a Word document and a PDF version with tracked changes.

REVIEWER #1

1. Introduction: axial diameter <95th percentile? please clarify. **The authors have modified the definition to clarify.**

2. Embriology (remove relevant): Add commas between "termed the embryonal/choroidal/fetal fissure". **The authors have modified the sentence.**

3. Pediatric examination: amend "It is also crucial". **The authors have changed the sentence to clarify.**

4. Ophthalmological assessment: remove the repetition "ranging". **The authors removed the repetition.**

5. Further investigations: please clearly include, among the defects identified through imaging investigations assessing CNS structures related to eyes, conditions like septo-optic dysplasia. **The authors have modified the sentence according to the suggestion.**

6. Imaging in prenatal diagnosis: improve the sentence "The absence of a lens...may complicate evaluation". **The authors modified the sentence to clarify.**

7. Molecular prenatal diagnosis: please consider and include non invasive investigations like cell fetal DNA in maternal blood. **The authors added a sentence according to the Reviewer’s suggestion and a relative reference.**

REVIEWER #2

The authors have followed the reviewers' recommendations and made the appropriate revisions to the text.

Additionally, if this modification does not hinder the publication of the article, the authors have included a sentence in Section “3. Etiology and Genetics”, regarding the association between ocular hypoplasia and nasal abnormalities. Accordingly, we have also added the gene in question to Table 2.

Furthermore, we received official Ethical approval and consent for publication of this Review. We have added this specification at the end of the manuscript.

Hoping we have adequately addressed the reviewers' requests; we remain at your disposal for any further revisions that may be needed.

Genova, January 8th, 2024 **Monica Russo**
