# Supplementary material for: Management of anophthalmia, microphthalmia and coloboma in the newborn, shared care between neonatologist and ophthalmologist: a literature review
Source: Ital J Pediatr. 2025 Mar 5;51:65. doi: 10.1186/s13052-025-01882-3 (PMC11881466; doi:10.1186/s13052-025-01882-3)
Supplement: Supplementary file 3 — Supplementary Material 3 [file 13052_2025_1882_MOESM3_ESM.pdf]

Management of anophthalmia, microphthalmia and coloboma in the newborn, shared care between neonatologist and ophthalmologist: a literature review

by Monica Russo[1], Serena Palmeri[2], Alice Zucconi[2], Aldo Vagge[3], Cesare Arioni[1]*

[1]Operative Unit of Neonatology, IRCCS Ospedale Policlinico San Martino, Genoa, Italy

[2]Department of Neurosciences, Rehabilitation, Ophthalmology, Genetics and Maternal and Child Sciences (DINOGMI), University of Genoa, Genoa, Italy

[3]Pediatric Ophthalmology and Strabismus University Eye Clinic of Genoa DiNOGMI, University of Genoa - IRCCS Ospedale Policlinico San Martino Genoa, Italy

***Corresponding Author:** Monica Russo, Operative Unit of Neonatology, IRCCS Ospedale Policlinico San Martino, Genoa, 16132, Italy; +39 010-5553736, monica.russo@hsanmartino.it

Short running title: Anophthalmia, microphthalmia, and coloboma in newborns

Abstract

Congenital ocular anomalies significantly contribute to global disability, with 15-20% of infant blindness attributed to these anomalies. This study examined anophthalmia, microphthalmia, and coloboma (AMC) through collaborative neonatology and ophthalmology care.

The global prevalence of AMC varies: anophthalmia at 0.6-4.2 per 100,000 births and microphthalmia at 2-17 per 100,000 births, with a combined prevalence of up to 30 per 100,000. The prevalence of coloboma, alone or associate with other eye defects is 2–19 per 100,000 live births. Anophthalmia and microphthalmia may present as isolated or genetic syndromes, necessitating comprehensive evaluation. AMC etiology encompasses genetic and environmental factors. Chromosomal aberrations and mutations in genes such as *PAX6*, *SOX2*, *OTX2*, and *CHD7* are contributors. Syndromic associations, such as CHARGE (heart defect, atresia choanae, retarded growth and development, genital hypoplasia, ear anomalies/deafness) syndrome, underscore the complexity of this syndrome. Early AMC diagnosis is pivotal for timely intervention. This work provides a literature review offering insights for effective management and genetic counseling in a pediatric context.

Keywords: anophthalmia, microphthalmia, coloboma, newborn, congenital eye malformation, care report

Abbreviations: *AMC,* Anophthalmia, microphthalmia, and coloboma; *CT,* Computed tomography; *FGF,* fibroblast growth factor; MRI, magnetic resonance imaging; *NGS*, next-generation sequencing; *OAEs*, Otoacoustic emissions; *RD*, retinal detachment*; RRT*, red-reflex test; *RT-PCR*, real-time polymerase chain reaction; *SHH*, sonic hedgehog; TGFβ, transforming growth factor

Main text

Introduction

Congenital ocular anomalies are a significant cause of disability worldwide and are estimated to be responsible for approximately 15% to 20% of blindness and severe visual impairment in infancy. (1)

Anophthalmia is defined as the complete absence of the eye globe in the orbit.
Microphthalmia refers to an underdeveloped eye of subnormal size, usually defined in terms of corneal diameter or axial length. (2,3) ~~An eye is termed microphthalmic when the axial diameter (adjusted for age)~~ An eye is termed microphthalmic when the axial diameter is <16 mm at birth, <18.5-19 mm in adults (and in children aged >1 year).~~is <95th percentile; in adults (and in children aged >13 years), an axial length <18.5-19 mm is considered microphthalmic. (4)~~(1) (4)

The term coloboma describes a segmental ocular defect in any ocular tissue consistent with failure of closure of the embryonal fissure. The embryonal fissure is located inferonasally and extends into a cleft on the lower surface of the optic stalk. Hence, embryonal fissure-related problems can involve, in addition to the choroid and retina, the optic disc posteriorly and the iris and ciliary body anteriorly.

“Typical” coloboma refers to a defect in the inferior/inferonasal part of the fundus that can be clearly attributed to a defect in the closure of the embryonal fissure. There is a rare subgroup of colobomas, referred to as "atypical," which are located in other parts of the eye (nasal, temporal, or superior), and their pathogenesis has not yet been clarified. Atypical coloboma appears to be sporadic, as no familial cases have been identified yet. (4) (5) (6)

Early detection of these malformations is important for providing early and more effective treatment and rehabilitation strategies and for providing genetic counseling, if appropriate.

We will provide a literature review on major structural eye malformations, such as anophthalmia, microphthalmia, and coloboma (AMC), along with appropriate multidisciplinary management in a pediatric contest.

1. Epidemiology. The prevalence of the main congenital anomalies of the eyeball (microphthalmos, anophthalmos, and coloboma) results in wide variability among different geographical areas. The birth prevalence of anophthalmia ranges from 0.6 to 4.2 per 100,000 births, from 2 to 17 per 100,000 births for microphthalmia, while the combined birth incidence has been reported to reach 30 per 100,000 people. (1) (2) (7) (8) (9)
The prevalence of coloboma, alone or associate to other eye defects is 2–19 per 100,000 live births.(5)

Anophthalmia and microphthalmia are bilateral in most cases, except for isolated microphthalmia, which is usually unilateral. (2) Both anophthalmia and microphthalmia can be isolated or associated with other ocular malformations or organ disease or can be part of a genetic syndrome. The most common associated congenital ocular anomaly is a coloboma in the same or contralateral eye. (10) The risk factors for these malformations suggested by epidemiological studies are maternal age over 40 years, multiple pregnancies, low birth weight and low gestational age. (2)

2. Relevant embryology Eye development occurs in human embryos between approximately the third and tenth weeks of gestation. Ocular tissues are mesodermal and ectodermal in origin. The retina, ciliary body, optic nerves, and iris derive from the neuroepithelium. The lens, eyelid, and corneal epithelium develop from the surface ectoderm. The sclera, blood vessels, ocular muscles, vitreous, corneal endothelium, and stroma develop from the extracellular mesenchyme. (5) The homeobox gene *PAX6* is essential for the initiation of the process, which begins with evagination of the optic grooves in the medial anterior neural plate. During the fourth week of pregnancy, upon neural tube closure, the optic grooves are transformed into optic vesicles through the forebrain. The optic vesicles invaginate to form a double-layered optic cup, the inner layer of which develops into the retinal pigment epithelium, while the outer layer forms the retinal pigment epithelium (RPE). The iris and ciliary body develop from the middle part of the optic cup. As the optic cup invaginates, the surface ectoderm forms the lens placode and then develops into the lens vesicle. (11) (12) A ventral invagination along the optic cup and optic stalk termed the embryonal~~/~~, choroidal,~~/~~fetal fissure permits the mesenchyme to enter the optic cup and form blood vessels. This fissure closes normally ~~by~~after 5–7 weeks of gestation (at approximately the 17-mm stage). (12) Several transcription factors, such as *PAX6*, *SIX3*, *LHX2*, and *RAX*, are needed at each stage of eye development. Extrinsic factors, including members of the transforming growth factor (TGFβ), fibroblast growth factor (FGF), sonic hedgehog (SHH), and WNT signaling pathway, are important regulators of ocular embryogenesis. (4) (11)

3. Etiology and genetics. The etiology of AMC is complex, and both genetic and environmental factors are involved. Genetic contributions are significant, and multiple genes have been identified in association with AMC. AMC can be caused by numerical chromosomal defects (duplications, deletions, or translocations) as well as by mutations in selected genes. Chromosomal aberrations are typically associated with characteristic syndromes, such as trisomy 13 (Patau syndrome) and trisomy 18 (Edwards syndrome), or with systemic anomalies in addition to ocular malformation. Rare but equally significant defects should be considered on a case-by-case basis. (13) (14)

Monogenic causes include variants in many single genes, including *PAX6*, *SOX2*, *OTX2* and *CHD7*. (2) (15) The mutations can be de novo or inherited. The main reported chromosomal abnormalities and single gene mutations associated with AMC are listed in Table 1 and Table 2, respectively.

In severe bilateral cases of anophthalmia and microphthalmia, a genetic cause has been identified in approximately 80% of cases, with de novo heterozygous loss-of-function point mutations in *SOX2* being the most common, accounting for 10-20% of cases. (2) (16) *SOX2*-associated ocular malformations, including anophthalmia, microphthalmia, sclerocornea, cataracts, persistent hyperplastic primary vitreous and optic disc dysplasia, are variable in type but are most often bilateral and severe. The phenotype of "SOX2 anophthalmia syndrome" includes extraocular features such as learning disabilities, facial dysmorphisms, postnatal growth failure, esophageal atresia with or without trachea-esophageal fistula and urogenital anomalies. (17) (18)

Although, as mentioned before, the *PAX6* gene is embryologically involved in eye development, its mutations are rare causes of anophthalmia/microphthalmia. Heterozygous loss-of-function (LOF) mutations of this gene, located on chromosome 11p13, are typically associated with aniridia, a congenital panocular malformation characterized by variable

gravity. (2)
Mutations in *OTX2*, *RAX* and *CHX10*, three genes expressed in the retina, are reportedly associated with anophthalmia/microphthalmia, possibly causing failure of retinal differentiation. (2) Heterozygous loss of function mutations in *OTX2* on chromosome 14q22 cause a wide variety of ocular anomalies, ranging from anophthalmia/microphthalmia to retinal defects, eventually associated with CNS malformations. (19) Mutations in *RAX*, located on chromosome 18q21.32, account for approximately 2% of inherited anophthalmia/microphthalmia (20) . Another 2% of isolated microphthalmia is attributed to mutations in the *CHX10* gene on chromosome 14q24.3, with autosomal recessive inheritance. (21) Mutations in MSCHD1 gene have been reported in individuals with eye hypoplasia and nose malformations. (22) (23)
AMC has also been described in the context of a genetic syndrome. Mutations in the *GLI2* gene were first reported in association with holoprosencephaly and polydactyly; subsequently, anophthalmia and orbital anomalies have also been incorporated into the phenotype. (24) Mutations in the *STRA6* gene cause a variable syndromic phenotype that includes anophthalmia, congenital heart defects and diaphragmatic hernia, pulmonary abnormalities and intellectual disability. (25)
The most common genetic syndrome associated with coloboma is CHARGE syndrome, an acronym that describes its wide range of clinical features, including coloboma, heart defects, choanal atresia, retardation (of growth and/or development), genitourinary malformation and ear abnormalities. In CHARGE syndrome, almost all patients have intrinsic ophthalmic defects in at least one eye. These include optic nerve/retinochoroidal coloboma, microphthalmia, cataract, and iris coloboma. (26) Currently, the only gene known to be implicated in CHARGE syndrome is *CHD7*, located on 8q12, which regulates the transcription of other tissue-specific targets and possibly disrupts neural crest migration when mutated. (27)
Another rare syndrome which can be associated with ocular defects is Cat Eye syndrome, named after the characteristic elongated shape of the pupil that may be present in this condition. The three most common features are the symptom triad of preauricular anomalies, anal atresia, and iris coloboma, though there is a very broad phenotypic range. It is typically caused by a partial tetrasomy of chromosome 22, which arises from a supernumerary dicentric marker chromosome featuring satellite structures at both ends and an inverted duplication of chromosome 22. (28)
Environmental factors are also implicated in the etiology of AMC. The strongest evidence of gestational-acquired infections is associated with syphilis, rubella, varicella, toxoplasmosis, cytomegalovirus, and other viruses, such as parvovirus B19, influenza virus, and coxsackie A9. (2) (29) (30) (31)
The principal environmental causes of congenital coloboma are reported in Table 3. (32)
Several noninfectious causes have been proposed, including vitamin A deficiency, maternal diabetes, hypothyroidism, maternal consumption of drugs such as thalidomide, carbamazepine, idantoin, warfarin, exposure to X-rays, and hyperthermia. (2) (4)
Notably, perinatal alcohol exposure can cause various ocular defects. Visible eye abnormalities in fetal alcohol syndrome include shortened and horizontal palpebral fissures, telecanthus, epicanthus, and blepharoptosis. Strabismus has also been reported. Intrinsic eye structure defects, which indicate early exposure to these teratogens, include microphthalmia, buphthalmos, iris and uveal coloboma, and retinal or vitreous malformations. (33)

4. Diagnosis and management. Usually, congenital ocular malformation is initially suspected by neonatologists based on clinical examination. Then, diagnosis and management require ophthalmological assessment and imaging. A comprehensive family and medical history, physical examination, instrumental and laboratory tests and genetic testing will be needed to establish a specific etiology, and then to provide appropriate counselling to families.

4.1. Pediatric examination. Clinical examination of the newborn by the neonatologist usually first raises the diagnostic suspicion at birth: inspection and palpation of the eye globe through the lids to confirm its presence and obtain an estimate of the ocular size is essential. The majority of cases are identified because of visible eye anomalies, such as a small eyeball, gross nystagmus, strabismus or an obvious iris coloboma. (4) (34) (35)

The red-reflex test (RRT) is a valuable tool for pediatricians for screening ocular anomalies: any factor that impedes, blocks, or changes this path of the light will result in an abnormal RRT. High and/or asymmetrical refractive defects and ocular misalignment (strabismus) can alter RRT. Anophthalmia and microphthalmia could determine asymmetrical or absent RRT. (34) (36)

Specifically, gross nystagmus and strabismus may subtend different and wide etiologies including neurological diseases, rather than ocular malformations.
Congenital nystagmus can be linked to several ocular and neurological conditions, or idiopathic. Common causes include Leber congenital amaurosis, albinism, aniridia, achromatopsia, and optic nerve hypoplasia. Other conditions such as optic atrophy, bilateral congenital cataracts, and congenital stationary night blindness may also contribute to its development. A thorough assessment is essential to identify the underlying cause in affected individuals and guide appropriate management strategies.
Congenital strabismus in newborns can be caused by damage to the eye muscles or the nerves that innervate them. Such damage may be associated with conditions like cerebral palsy or, less commonly, acute vascular injury. The clinician should carefully exclude any trauma that may have occurred during the peripartum period.~~It's also crucial to carefully exclude any trauma that may have occurred during the peripartum period.~~ (37)

A complete physical examination is recommended to identify any associated dysmorphic features or malformations.

4.2. Ophthalmological assessment. Once the suspicion of a congenital ocular anomaly has been established, a specialist ophthalmological assessment is needed to study the transparency of the dioptric apparatus, examine the fundus, and measure the corneal diameters and axial length.
Anophthalmia can be a difficult diagnosis to make by clinical examination. In some cases, with no clinical evidence of a globe or ocular tissue, residual neuroectoderm was demonstrated on histological samples; hence, the use of terms such as “clinical anophthalmia” and “extreme microphthalmia” may refer to a phenotypic spectrum ranging ~~ranging~~ from anophthalmia to microphthalmia. (2)
Microphthalmia is diagnosed by measuring the corneal diameter, which ranges from 9–10.5 mm in neonates and 10.5–12 mm in adults. Nevertheless, corneal diameter cannot always be used as a surrogate marker of eye dimension, as normal-sized eyeballs can also present with microcornea. An ophthalmologist can more precisely measure the axial length of the eye via

ultrasonography. (2)
The diagnosis of a coloboma and its extent require accurate evaluation of both the anterior and posterior segments of the eye. Iris involvement is often observed in association with fundus coloboma. A complete iris typical coloboma appears as an inferonasal defect that merges with the pupil in the shape of a keyhole, while an incomplete iris coloboma can be seen as a notch in the inferior pupillary border or a defect in the pigment epithelium or heterochromia. In contrast to traumatic iris defects, the margins of a coloboma are smooth. A lens coloboma can be seen in a dilated eye as the equator of the lens flattens in an area without zonular fibers. In fundus coloboma, the choroid and retinal pigment epithelium are absent and appear as a white area of bare sclera with occasional spots of pigment deposition at the junction with the normal retina; the border can be smooth or scalloped. Its extent is variable, as it can reach and invade the periphery or be restricted to islands along a line joining disc with an inferior/inferonasal periphery. Bridge coloboma is a term used to describe two islands of colobomas interspersed with a normal retina. The examiner should evaluate for retinal detachment (RD), as patients with colobomas have an increased risk of RD during their lifetime. (4)
A thorough examination of the family history, focusing on the presence of ocular anomalies, along with ophthalmological assessments of both parents, should be conducted.

4.3. Imaging. Eye ultrasound is recommended for accurate determination of the axial length of the globe and for evaluation of internal ocular structures. (2)
Computed tomography (CT) and magnetic resonance imaging (MRI) of the orbits can aid clinicians in the diagnosis of anophthalmia, demonstrating the absence of ocular tissue within the orbit, which is usually associated with reduced orbital dimensions; residual optic nerve neural tissue and extraocular muscles are variable. The microphthalmic eye appears on CT and MRI scans as an eye globe of reduced dimensions with normal density/signal intensity of lens and vitreous, usually in a smaller orbit. In addition, orbital CT or MRI scan may show the presence of a cyst posterior to the eyeball which can sometimes be associated with microphthalmos. (2) (38)

4.4. Further investigations. Further investigations may be needed depending on the clinical picture. Brain MRI allows the study of the intracerebral optic pathways (optic nerves, optic chiasm, optic tracts, optic radiations) and potentially associated cerebral malformations, such as septo-optic dysplasia, a rare congenital disorder characterized by the triad of: a) optic nerve hypoplasia, b) midline developmental defects including agenesis of the septum pellucidum, agenesis or dysgenesis of the corpus callosum, or both, and c) anomalies of the hypothalamic-pituitary axis. This malformation is reported in association with bilateral anophthalmia/microphthalmia.~~Further investigations may be needed depending on the clinical picture. Brain MRI allows the study of the intracerebral optic pathways (optic nerves, optic chiasm, optic tracts, optic radiations) and potentially associated cerebral malformations, such as agenesis or dysgenesis of the corpus callosum, which is frequently reported in association with bilateral anophthalmia.~~ (39)
In a study conducted by Huynh et al. on 99 patients with apparently isolated uveal coloboma, abnormal findings were detected via echocardiography (53%, 10 of 19 patients who underwent echocardiography; ventral septal defects were the most prevalent), brain MRI (17%, 5 of 29 patients), audiology testing (17%, 13 of 75 patients), spine X-ray (13%, 10 of 77 patients) and kidney US (7%, 5 of 72 patients). Therefore, the authors suggest a protocol

for the evaluation of seemingly isolated uveal colobomas, which includes physical examination, baseline audiology assessment, renal US and spine radiography. (40)

5. Prenatal diagnosis. Prenatal diagnosis of AMC has become increasingly important for early intervention and management.

5.1 Imaging in prenatal diagnosis. The prenatal diagnosis of AMC is increasingly important for early intervention and management. Various imaging techniques, such as ultrasound and fetal MRI, are used to detect coloboma-related anomalies during pregnancy. Intrauterine MRI can identify eye malformations like anophthalmia and microphthalmia, as well as nervous tissue abnormalities associated with coloboma. However, isolated prenatal diagnoses of coloboma using MRI have been reported. (41) (42)

The use of ultrasound for diagnosis of fetal ocular defects was first described in 1991 (43). Orbital imaging should begin at 12 weeks' gestation, with detailed eye examinations playing a crucial role in diagnosing microphthalmia or anophthalmia. This helps with genetic counseling, postnatal care planning, and parental preparation, including discussions on pregnancy termination. Two-dimensional ultrasound may show an absence of the eye globe and lens, while three-dimensional reverse-face imaging, as described by Araujo et al. in 2012, can confirm the diagnosis and reveal additional features, such as sunken eyelids and hypoplastic orbits, even when fetal head position interferes with 2D imaging (44). Three-dimensional reverse-face imaging may reveal valuable additional sonographic features, including sunken eyelids and small or hypoplastic orbit on the affected side and may be considered even superior to 2D when fetal head is deviated. The absence of a lens (aphakia) or the presence of hyaloid arteries should alert clinicians to potential eye abnormalities. These conditions may be associated with complications such as orbital cysts or hemangiomas, which can hinder accurate evaluation.~~The absence of a lens (aphakia) or the presence of hyaloid arteries should raise suspicion of eye abnormalities, as orbital cysts or hemangiomas may complicate evaluation.~~ (45)

Fetal MRI complements ultrasound by providing detailed insights into central nervous system malformations and confirming the absence of eye tissue, optic nerves, and extraocular muscles in cases of anophthalmia . (46)

5.2. Molecular prenatal diagnosis. Invasive prenatal diagnostics offer the opportunity for genetic diagnosis before birth through the analysis of fetal cells collected via procedures such as amniocentesis (usually after 14 weeks of gestation) or chorionic villus sampling (from 10 to 12 weeks of gestation). Among the applicable cytogenetic tests are karyotyping, chromosomal microarray (including SNP array and CGH array), and targeted next generation sequencing (NGS) panels. In case of suspect of a genetic condition, postnatal cord blood testing is also recommended. Non invasive prenatal investigations include genetic testing using cell-free fetal DNA (cffDNA) present in maternal blood. (46) (47) The prenatal genetic workup should be planned in consultation with a geneticist, considering the fetus's malformative characteristics and family history.

6. Treatment

In managing microphthalmia/anophthalmia, the primary aim is to support optimal visual function development, depending on the disease's severity and the ocular structures' integrity and developmental capacity.

The treatment of congenital coloboma includes various medical and surgical interventions. Medical treatment primarily involves addressing associated vision impairments and promoting visual development in affected individuals. This can be achieved through the early prescription of corrective lenses, occlusion therapy to improve binocular vision, and regular monitoring by ophthalmologists specializing in pediatric eye care

The proper development of orbital cavity and craniofacial conformation is achieved whenever needed through surgically assisted cavity expansion with socket expansion and eye conformers. Given the often genetic etiology of this condition, genetic counseling is essential, allowing for coordinated referrals to relevant specialists for comprehensive care. (2) (48) (49) (50)

Surgical management for the craniofacial development is pivotal for the correct psychological development of young patients, as those affected by forms of hemifacial microsomia are usually affected by higher risk of behavior problems, social difficulties and less acceptance. Therefore, early implementation of eye prothestetics and eye conformers is recommended. (51) (52)

It is important to note that the appropriateness and timing of surgical interventions depend on individual cases and should be determined by a multidisciplinary team comprising ophthalmologists, geneticists, and pediatric surgeons. (1) (9)

7. Communication with family and support. Since the neonatologist is the first doctor to examine the baby after birth, it is essential to establish effective communication with the family and to convey any suspected and/or confirmed diagnoses under the best possible conditions. The presence of the various professionals involved in managing the clinical case would be desirable, as would avoiding a short duration for the first meeting or an uncomfortable environment. (53)

Conclusion. The diagnosis of congenital coloboma, whether isolated or associated with additional clinical issues, is challenging due to the wide range of environmental and genetic causes. Recent advancements in prenatal radiological diagnostics and genetic counseling have enabled early identification of familial cases, promoting timely intervention. However, for all other cases, an early diagnosis through prompt examination of the newborn's eyes by a neonatologist and a coordinated approach between the neonatologist and ophthalmologist remain essential. Neonatologists can coordinate the multidisciplinary input needed to offer optimal care for newborns. (54)

Declarations

Ethical approval and consent for publication: Ethical clearance for the publication of this review was obtained from the Regional Ethical Commitee at the data 20/12/2024. Registration Number CET - Liguria: 447/2024 - DB id 14236**Ethics approval and consent to participate:** not applicable

Consent for publication: not applicable

Availability of data and materials: not applicable

Competing interest: The authors declare that they have no competing interests.

Funding: This publication received no external funding.

Authors' contributions: MR, AV, and CA conceived the idea, MR, AZ, and SP collected the data and wrote the manuscript, while CA, AV, and MR revised the manuscript.

Acknowledgments: Not applicable

Number of tables: 3

References

1. Shah SP, Taylor AE, Sowden JC, Ragge NK, Russell-Eggitt I, Rahi JS, et al. Anophthalmos, Microphthalmos, and Typical Coloboma in the United Kingdom: A Prospective Study of Incidence and Risk. Investig Opthalmology Vis Sci. 2011 Jan 31;52(1):558.

2. Verma AS, FitzPatrick DR. Anophthalmia and microphthalmia. Orphanet J Rare Dis. 2007 Dec;2(1):47.

3. Skalicky SE, White AJR, Grigg JR, Martin F, Smith J, Jones M, et al. Microphthalmia, anophthalmia, and coloboma and associated ocular and systemic features: understanding the spectrum. JAMA Ophthalmol. 2013 Dec;131(12):1517–24.

4. Lingam G, Sen AC, Lingam V, Bhende M, Padhi TR, Xinyi S. Ocular coloboma—a comprehensive review for the clinician. Eye. 2021 Aug;35(8):2086–109.

5. Yoon KH, Fox SC, Dicipulo R, Lehmann OJ, Waskiewicz AJ. Ocular coloboma: Genetic variants reveal a dynamic model of eye development. Am J Med Genet C Semin Med Genet. 2020 Sep;184(3):590–610.

6. Bacci GM, Polizzi S, Mari F, Conti V, Caputo R, Guerrini R. Atypical Ocular Coloboma in Tuberous Sclerosis-2: Report of Two Novel Cases. J Neuroophthalmol. 2021 Sep;41(3):e363.

7. Clementi M, Tenconi R, Bianchi F, Botto L, Calabro A, Calzolari E, et al. Congenital eye malformations: a descriptive epidemiologic study in about one million newborns in Italy. Birth Defects Orig Artic Ser. 1996;30(1):413–24.

8. Shaw GM, Carmichael SL, Yang W, Harris JA, Finnell RH, Lammer EJ. Epidemiologic characteristics of anophthalmia and bilateral microphthalmia among 2.5 million births in California, 1989-1997. Am J Med Genet A. 2005 Aug 15;137A(1):36–40.

9. Morrison D. National study of microphthalmia, anophthalmia, and coloboma (MAC) in Scotland: investigation of genetic aetiology. J Med Genet. 2002 Jan 1;39(1):16–22.

10. Fahnehjelm C, Dafgård Kopp E, Wincent J, Güven E, Nilsson M, Olsson M, et al. Anophthalmia and microphthalmia in children: associated ocular, somatic and genetic morbidities and quality of life. Ophthalmic Genet. 2022 Mar 4;43(2):172–83.

11. Mathers* PH, Jamrich M. Regulation of eye formation by the Rx and pax6 homeobox genes: Cell Mol Life Sci. 2000 Mar;57(2):186–94.

12. Ludwig PE, Lopez MJ, Czyz CN. Embryology, Eye Malformations. In: StatPearls [Internet]. Treasure Island (FL): StatPearls Publishing; 2023 [cited 2023 Jun 6]. Available from: http://www.ncbi.nlm.nih.gov/books/NBK482496/

13. Piro E, Serra G, Giuffrè M, Schierz IAM, Corsello G. 2q13 microdeletion syndrome: Report on a newborn with additional features expanding the phenotype. Clin Case Rep. 2021;9(6):e04289.

14. Serra G, Antona V, Giuffrè M, Piro E, Salerno S, Schierz IAM, et al. Interstitial deletions of chromosome 1p: novel 1p31.3p22.2 microdeletion in a newborn with craniosynostosis, coloboma and cleft palate, and review of the genomic and phenotypic profiles. Ital J Pediatr. 2022 Mar 4;48(1):38.

15. Bardakjian TM, Schneider A. The genetics of anophthalmia and microphthalmia: Curr Opin Ophthalmol. 2011 Sep;22(5):309–13.

16. Williamson KA, FitzPatrick DR. The genetic architecture of microphthalmia, anophthalmia and coloboma. Eur J Med Genet. 2014 Aug;57(8):369–80.

17. Ragge NK, Lorenz B, Schneider A, Bushby K, de Sanctis L, de Sanctis U, et al. SOX2 anophthalmia syndrome. Am J Med Genet A. 2005;135A(1):1–7.

18. Williamson KA, Hever AM, Rainger J, Rogers RC, Magee A, Fiedler Z, et al. Mutations in SOX2 cause anophthalmia-esophageal-genital (AEG) syndrome. Hum Mol Genet. 2006 May 1;15(9):1413–22.

19. Ragge NK, Brown AG, Poloschek CM, Lorenz B, Henderson RA, Clarke MP, et al. Heterozygous Mutations of OTX2 Cause Severe Ocular Malformations. Am J Hum Genet. 2005 Jun;76(6):1008–22.

20. Voronina VA. Mutations in the human RAX homeobox gene in a patient with anophthalmia and sclerocornea. Hum Mol Genet. 2003 Dec 8;13(3):315–22.

21. Ferda Percin E, Ploder LA, Yu JJ, Arici K, Jonathan Horsford D, Rutherford A, et al. Human microphthalmia associated with mutations in the retinal homeobox gene CHX10. Nat Genet. 2000 Aug;25(4):397–401.

22. Gordon CT, Xue S, Yigit G, Filali H, Chen K, Rosin N, et al. De novo mutations in SMCHD1 cause Bosma arhinia microphthalmia syndrome and abrogate nasal development. Nat Genet. 2017 Feb;49(2):249–55.

23. Russo M, Ferrecchi C, Rebella S, et al. Congenital Nasal Bones Agenesis: Report of a Rare Malformation. Case Rep Med. 2024;2024:1849957. Published 2024 Dec 23. doi:10.1155/carm/1849957Congenital Nasal Bones Agenesis: Report of a Rare Malformation - Russo - 2024 - Case Reports in Medicine - Wiley Online Library [Internet]. [cited 2025 Jan 10]. Available from: https://onlinelibrary.wiley.com/doi/10.1155/carm/1849957

24. Bertolacini C, Ribeiro-Bicudo L, Petrin A, Richieri-Costa A, Murray J. Clinical findings in patients with GLI2 mutations - phenotypic variability. Clin Genet. 2012 Jan;81(1):70–5.

25. Pasutto F, Sticht H, Hammersen G, Gillessen-Kaesbach G, FitzPatrick DR, Nürnberg G, et al. Mutations in STRA6 Cause a Broad Spectrum of Malformations Including Anophthalmia, Congenital Heart Defects, Diaphragmatic Hernia, Alveolar Capillary Dysplasia, Lung Hypoplasia, and Mental Retardation. Am J Hum Genet. 2007 Mar;80(3):550–60.

26. Kanwar K, Bashey S, Bohnsack BL, Drackley A, Ing A, Rahmani S, et al. Ocular manifestations of CHARGE syndrome in a pediatric cohort with genotype/phenotype analysis. Am J Med Genet A. 2024;194(8):e63618.

27. Hsu P, Ma A, Wilson M, Williams G, Curotta J, Munns CF, et al. CHARGE syndrome: A review: A review of CHARGE syndrome. J Paediatr Child Health. 2014 Jul;50(7):504–11.

28. Gaspar NS, Rocha G, Grangeia A, Soares HC. Cat-Eye Syndrome: A Report of Two Cases and Literature Review. Cureus. 2022 Jun 25;14(6):e26316.

29. Busby A, Dolk H, Armstrong B. Eye Anomalies: Seasonal Variation and Maternal Viral Infections. Epidemiology. 2005 May;16(3):317–22.

30. Givens KT, Lee DA, Jones T, Ilstrup DM. Congenital rubella syndrome: ophthalmic manifestations and associated systemic disorders. Br J Ophthalmol. 1993 Jun 1;77(6):358–63.

31. Kodjikian L, Wallon M, Fleury J, Denis P, Binquet C, Peyron F, et al. Ocular manifestations in congenital toxoplasmosis. Graefes Arch Clin Exp Ophthalmol. 2006 Jan;244(1):14–21.

32. Selzer EB, Blain D, Hufnagel RB, Lupo PJ, Mitchell LE, Brooks BP. Review of evidence for environmental causes of uveal coloboma. Surv Ophthalmol. 2022 Jul;67(4):1031–47.

33. Strömland K, Pinazo-Durán MD. OPHTHALMIC INVOLVEMENT IN THE FETAL ALCOHOL SYNDROME: CLINICAL AND ANIMAL MODEL STUDIES. Alcohol Alcohol. 2002 Jan 1;37(1):2–8.

34. Committee on Practice and Ambulatory Medicine, Section on Ophthalmology. American Association of Certified Orthoptists, American Association for Pediatric Ophthalmology and Strabismus, American Academy of Ophthalmology. Eye examination in infants, children, and young adults by pediatricians. Pediatrics. 2003 Apr;111(4 Pt 1):902–7.

35. Mehner LC, Singh JK. Ocular Disorders in the Newborn. NeoReviews. 2021 Jul 1;22(7):e461–9.

36. Taksande A, Jameel P, Taksande B, Meshram R. Red reflex test screening for neonates: A systematic review and meta analysis. Indian J Ophthalmol. 2021;69(8):1994.

37. Mocan MC, Pastapur A, Kaufman L. Etiology-based strabismus classification scheme for pediatricians. Turk J Pediatr. 2022 Apr 25;64(2):332–40.

38. Ragge NK, Subak-Sharpe ID, Collin JRO. A practical guide to the management of anophthalmia and microphthalmia. Eye. 2007 Oct;21(10):1290–300.

39. Albernaz VS, Castillo M, Hudgins PA, Mukherji SK. Imaging Findings in Patients with Clinical Anophthalmos. 1997;

40. Huynh N, Blain D, Glaser T, Doss EL, Zein WM, Lang DM, et al. Systemic Diagnostic Testing in Patients With Apparently Isolated Uveal Coloboma. Am J Ophthalmol. 2013 Dec;156(6):1159-1168.e4.

41. Brémond-Gignac D, Copin H, Elmaleh M, Milazzo S. Anomalies oculaires fœtales : apport de l'imagerie anténatale en résonance magnétique. J Fr Ophtalmol. 2010 May;33(5):350–4.

42. Egloff C, Tassin M, Bault JP, Barjol A, Collin A, Simon I, et al. Prenatal description of retinal coloboma. J Gynecol Obstet Hum Reprod. 2020 Sep;49(7):101746.

43. Bronshtein M, Zimmer E, Gershoni-Baruch R, Yoffe N, Meyer H, Blumenfeld Z. First- and second-trimester diagnosis of fetal ocular defects and associated anomalies: report of eight cases. Obstet Gynecol. 1991 Mar;77(3):443–9.

44. Wong HS, Parker S, Tait J, Pringle KC. Antenatal diagnosis of anophthalmia by three-dimensional ultrasound: a novel application of the reverse face view. Ultrasound Obstet Gynecol. 2008;32(1):103–5.

45. Varejão AM, Pestana I. Prenatal diagnosis of isolated bilateral anophthalmia. BMJ Case Rep CP. 2021 Aug 1;14(8):e244684.

46. Searle A, Shetty P, Melov SJ, Alahakoon TI. Prenatal diagnosis and implications of microphthalmia and anophthalmia with a review of current ultrasound guidelines: two case reports. J Med Case Reports. 2018 Aug 29;12(1):250.

47. Harding P, Brooks BP, FitzPatrick D, Moosajee M. Anophthalmia including next-generation sequencing-based approaches. Eur J Hum Genet. 2020 Mar;28(3):388–98.

48. Harding P, Gore S, Malka S, Rajkumar J, Oluonye N, Moosajee M. Real-world clinical and molecular management of 50 prospective patients with microphthalmia, anophthalmia and/or ocular coloboma. Br J Ophthalmol. 2023 Dec 1;107(12):1925–35.

49. Changal N, Khandekar RB. Eye Conformers as Socket Expanders in Children: Experience at a Tertiary Eye Hospital in Central Saudi Arabia. Cureus. 2021 Feb 21;13(2):e13465.

50. Watanabe A, Singh S, Selva D, Tong JY, Ogura T, Kajiyama S, et al. Socket expansion with conformers in congenital anophthalmia and microphthalmia. J AAPOS Off Publ Am Assoc Pediatr Ophthalmol Strabismus. 2022 Dec;26(6):318.e1-318.e6.

51. Paul MA, Opyrchał J, Knakiewicz M, Jaremków P, Bajtek J, Chrapusta A. Hemifacial Microsomia Review: Recent Advancements in Understanding the Disease. J Craniofac Surg. 2020 Dec;31(8):2123.

52. Mattos BSC, Montagna MC, Fernandes C da S, Sabóia ACL. The pediatric patient at a maxillofacial service: eye prosthesis. Braz Oral Res. 2006 Sep;20:247–51.

53. Serra G, Memo L, Coscia A, Giuffré M, Iuculano A, Lanna M, et al. Recommendations for neonatologists and pediatricians working in first level birthing centers on the first communication of genetic disease and malformation syndrome diagnosis: consensus issued by 6 Italian scientific societies and 4 parents' associations. Ital J Pediatr. 2021 Apr 19;47(1):94.

54. Serra G, Giuffrè M, Piro E, Corsello G. The social role of pediatrics in the past and present times. Ital J Pediatr. 2021 Dec 18;47(1):239.
