# Supplementary material for: Management of anophthalmia, microphthalmia and coloboma in the newborn, shared care between neonatologist and ophthalmologist: a literature review
Source: Ital J Pediatr. 2025 Mar 5;51:65. doi: 10.1186/s13052-025-01882-3 (PMC11881466; doi:10.1186/s13052-025-01882-3)
Supplement: Supplementary file 2 — Supplementary Material 2 [file 13052_2025_1882_MOESM2_ESM.pdf]

Dear Editors and Reviewers,

We sincerely thank you for the opportunity to revise our manuscript ITJP-D-24-00196, initially titled “Management of anophthalmia, microphthalmia and coloboma in the newborn, shared care between neonatologist and ophthalmologist: a case report and literature review.”
We appreciate the time and effort you have dedicated to providing insightful comments and suggestions to improve the quality of our work. In response, we have carefully addressed each point and have made the necessary revisions to strengthen the manuscript.

Below, we outline how each comment has been addressed in detail. Please see below the bolded sections with the recommended revisions.

Attached as separate documents are the file with track changes and the revised manuscript.

Reviewer reports:

Reviewer #1: Congratulations to the authors for the interesting work done.

The paper appears flowing and quite exaustive, but it could be appropriate if considered as a review article, rather than a case report. Indeed, the case presentation is scarsely contributive and not complete, poorly suitable to underline the diagnostic path and collaboration among different medical specialties when dealing with cases of AMC. How is it possible to provide genetic counselling without the results of investigations like a-CGH or NGS conducted in the whole trio? It does not emerge, from the description of the report, the diagnostic path followed in the suspicion of specific malformation syndromes/genetic diseases, such as for example Waardenburg syndrome, septo-optic dysplasia. Therefore it should be removed. **We removed the case report as indicated and, accordingly, we modified the abstract and the title in “Management of anophthalmia, microphthalmia and coloboma in the newborn, shared care between neonatologist and ophthalmologist: a literature review”**

Moreover, to enrich the purely review section:

a brief reference to Charge and Cat eye syndromes, whose ocular involvement is predominant within the clinical picture, should be made and added by authors; **The authors have added a brief reference on both CHARGE and Cat Eye syndrome in the section 3 Etiology and genetics.**

a consideration about prenatal genetic investigations, including a-CGH and NGS analysis techniques, should be included; **The authors have included a detailed section about prenatal genetic investigations.**

please argue about the management of microphthalmia and anophthalmia, not currently considered in the description of treatments. **We have integrated the manuscript with additional details on the management of microphthalmia and anophtalmia.**

Among minor revisions, please consider the following suggestions:

Abstract

prevalence of coloboma is not mentioned, please include it; **The authors have included a statement on the prevalence of coloboma.**

"may present as isolated or within genetic syndromes..." **The authors have modified.**

"and mutations in genes such as ...and CHD7 (please write them in italics) have a key role in the pathogenesis **The authors have modified.**

please remove the reference to International Clearinghouse for Birth defects monitoring systems, as it refers to adults classification, and then poorly pertinent to the argument here treated **The authors have modified.**

Epidemiology

please do not repeat in this section the same concepts expressed in the introduction. **The authors ensured that there were no repetitions in the two sections.**

Etiology and genetics

"congenital heart defects and diaphragmatic hernia, pulmonary abnormalities and intellectual disability" **The authors have revised the sentence.**

Among congenital infections please add syphilis, and among non-infectious diseases argue better and deepen about the role and importance of alcohol spectrum disorders, also in light of its high epidemiological and clinical impact. **The authors have added the suggested section and related reference.**

Diagnosis and management

"physical exmination, instrumental and laboratory exams and genetic testing will be needed to establish a specific etiology, and then to provide appropriate counselling to families **The authors have changed the sentence as suggested.**

Pediatric examination

gross nystagmus and strabismus may subtend different and wide etiologies inlcuding neurological diseases, rather than ocular malformations; please specify **The authors have completed the section as requested.**

Ophthalmological assessment

"..and "extreme microphthalmia" may refer to a phenotypic spectrum ranging..." **The authors have revised the sentence.**

"optic radiations, and potentially associated cerebral malformations, such as..." **The authors revised the sentence.**

"In a study conducted...on 99 patients..." **“Of” has been changed in “on”.**

Prenatal diagnosis

"Prenatal diagnosis of this condition"? Do the authors refer to AMC? please clarify **The authors have clarified this point.**

In regard with the genomic rearrangements associated with ocular malformations including microphthalmia (or epibulbar cysts), the authors should consider and properly add in the corresponding part of the text the following references:

2q13 microdeletion syndrome: report on a newborn with additional features expanding the phenotype. Ettore Piro,, Gregorio Serra,, Mario Giuffrè, Ingrid Anne Mandy Schierz, Giovanni Corsello. Clin Case Rep. 2021;9:e04289.

Interstitial deletions of chromosome 1p: novel 1p31.3p22.2 microdeletion in a newborn with craniosynostosis, coloboma and cleft palate, and review of the genomic and phenotypic profiles. Gregorio Serra, Vincenzo Antona, Mario Giufrè, Ettore Piro, Sergio Salerno, Ingrid Anne Mandy Schierz and Giovanni Corsello. Ital J Pediatr 2022 Mar; 8:38.

The authors added the suggested reference to the section "Etiology and genetics".

In relation to the management of these newborns, within the multidisciplinary care, the authors should consider and mention the high psychological impact of congenital defects like AMC. About this point please consider and add the following manuscript:

Recommendations for neonatologists and pediatricians working in first level birthing centers on the first communication of genetic disease and malformation syndrome diagnosis: consensus issued by 6 Italian scientific societies and 4 parents' associations. Serra G, Memo L, Coscia A, Giuffrè M, Iuculano A, Lanna M, Valentini D, Contardi A, Filippeschi S, Frusca T, Mosca F, Ramenghi LA, Romano C, Scopinaro A, Villani A, Zampino G, and Corsello G, on behalf of their respective Scientific Societies and Parents' Associations. Ital J Pediatr 2021 Apr 19;47:94. **The authors have added a new section to the manuscript "7. Communication with family and support".**

Moreover, with respect to the improvements achieved by the more recent and advanced surgical techniques along with the next generation and molecular investigation analyses, usuful for a better expectancy and quality of life of patients and their families (such concept must be included among the conclusive considerations), please add the following publication:

The social role of pediatrics in the past and present times. Gregorio Serra, Mario Giuffrè, Ettore Piro and Giovanni Corsello. Ital J Pediatr 2021 Dec;47: 239. **The authors have added the suggested reference.**

To make Table 2 more reader-friendly, please split it in three parts, connecting the genes listed with the 3 different clinical conditions studied **The authors, unless it will be limitant for publication, prefer to maintain the current structure of the table, giving the list of genes in alphabetical order. This consideration is due to the mixed phenotype of certain conditions, which could cause a repetition in the table.**

Reviewer #2: *Some revisions are necessary*

- *Line 30 page 4 insert new ref : "Atypical Ocular Coloboma in Tuberous Sclerosis-2: Report of Two Novel Cases G. M. Bacci, S. Polizzi, F. Mari, V. Conti, R. Caputo and R. Guerrini J Neuroophthalmol 2020"* **The authors have had the suggested reference.**

- *Line 54 pag 7 change "ocular diameter" with "corneal diameters and axial length"* **The authors have modified.**

- *Line 9 pag 8 change "complete coloboma" with "complete typical coloboma"* **The authors have modified.**
- *Line 39 pag 8 add a sentence like "Orbital CT o MRI scan may show the presence of a cyst posterior to the eyeball that can sometimes be associated with microphthalmos"* **The authors completed with the sentence.**
- *Lines 14-20 pag 9: I would remove the sentences regarding cosmetic surgery because it is extremely rarely adopted. Sometimes surgery on associated cataract or glaucoma can be performed especially in bilateral cases.. Instead, I would describe the importance of the early use of prostheses that is necessary to improve the development of orbit and eyelid size in eyes with absent visual capabilities* **The authors have removed the sentence as suggested and made the recommended revision.**

In accordance with Reviewer 1's request, we have removed the case report and retained the literature review. Therefore, we kindly ask the Editor to change the article type to "Review."

We remain available for any further requested modifications.

Genoa, 31/10/2024

Monica Russo
